# Associations Between the Severity of Sarcopenia and Health-Related Quality of Life in Older Adults

**DOI:** 10.3390/jcm15010161

**Published:** 2025-12-25

**Authors:** Wei-Syun Hung, Ying-Jen Chen, Tz-Shiu Tsai, Chern-Horng Lee, Ji-Tseng Fang, Ming-Shien Wen, Chun-Yen Lin, Kuo-Chen Liao, Chieh-Li Yen

**Affiliations:** 1 Geriatric Medical Center, Linkou Medical Center, Chang Gung Memorial Hospital, Taoyuan 333, Taiwan; weisyunhung@cgmh.org.tw (W.-S.H.); maxwell@cgmh.org.tw (T.-S.T.);; 2 Department of Geriatrics and General Internal Medicine, Linkou Medical Center, Chang Gung Memorial Hospital, Taoyuan 333, Taiwan; 3 Department of Chest Medicine, Linkou Medical Center, Chang Gung Memorial Hospital, Taoyuan 333, Taiwan; 4 Division of Hepatology, Department of Gastroenterology and Hepatology, Linkou Medical Center, Chang Gung Memorial Hospital, Taoyuan 333, Taiwan; 5 Department of Nephrology, Linkou Medical Center, Chang Gung Memorial Hospital, Taoyuan 333, Taiwan; 6 Department of Cardiology, Linkou Medical Center, Chang Gung Memorial Hospital, Taoyuan 333, Taiwan; 7 College of Medicine, Chang Gung University, Taoyuan 333, Taiwan

**Keywords:** sarcopenia, sarcopenia severity, quality of life, health-related quality of life, elderly

## Abstract

**Background:** Sarcopenia is a progressive skeletal muscle disorder associated with adverse outcomes. Although the association between sarcopenia and quality of life (QoL) has been discussed, the specific relationship between different stages of sarcopenia severity—particularly distinguishing between muscle mass loss and functional impairment—and health-related quality of life (HRQoL) remains unclear. **Method:** This cross-sectional study enrolled 100 elderly participants from a geriatric outpatient clinic. Participants were categorized into four groups (normal, possible sarcopenia, sarcopenia and severe sarcopenia) based on the 2019 Asian Working Group for Sarcopenia (AWGS) criteria. HRQoL was assessed using the Short-Form 36-Item (SF-36) questionnaire. **Result:** The severe sarcopenia group was significantly older and had lower calf circumference compared to the normal group. Notably, the possible sarcopenia group presented with the highest body mass index and body fat percentage, resembling a “dynapenic obesity” phenotype. In terms of QoL, participants with confirmed sarcopenia did not exhibit significant differences compared to the normal group. However, the severe sarcopenia group demonstrated significantly lower scores across almost all SF-36 domains compared to the normal group. Multivariate linear regression analysis revealed that severe sarcopenia was independently and significantly negatively associated with multiple QoL domains, including physical functioning, general health and vitality. Additionally, age, social activity and body fat were identified as independent correlates of specific QoL domains. **Conclusions:** Our findings suggest a non-linear relationship between sarcopenia and HRQoL. A comprehensive decline in HRQoL is strongly linked to the severity of sarcopenia (functional impairment) rather than the diagnosis of muscle mass loss alone. These results highlight the clinical importance of preserving physical performance and suggest that categorizing different severities of sarcopenia and stage-specific management strategies are necessary to improve quality of life in older adults.

## 1. Introduction

Gradual degradation of musculoskeletal functions is known as a part of the normal aging process. A previous study reported that a decrease in muscle mass (~1% per year) and strength (~2.5–3% per year) could be observed from the age of 60 [1]. However, sarcopenia is not merely a part of this process. It was first defined by the European Work Group on Sarcopenia in Older People (EWGSOP) in 2010 and was regarded as a geriatric syndrome [2]. In 2019, the definition was updated to describe sarcopenia as a “progressive and generalized skeletal muscle disorder associated with an increased likelihood of adverse outcomes, including falls, fractures, physical disability and mortality” [3]. Meanwhile, the operational definition of sarcopenia is also revised in 2019 EWGSOP, emphasizing that muscle strength is a better predictor of adverse outcomes than muscle mass. In addition, various expert groups worldwide, including those in Asia, have proposed complementary definitions of sarcopenia to address regional differences [4,5]. It is evident that the issue of sarcopenia has gained increasing attention alongside the rapid aging of the global population.

Indeed, sarcopenia has become one of the most common issues among the elderly population. In Taiwan, the prevalence of sarcopenia among community-dwelling elderly individuals varies from 3.9% to 16.6% (5.4–14.9% in men and 2.5–19% in women), depending on whether the EWGSOP or Asian Working Group for Sarcopenia (AWGS) criteria are used. Sarcopenia is not only associated with fall-related injuries [6], a large amount of researches have proved its link to adverse outcomes in various diseases, including numerous cancers [7,8], cardiovascular disease [9] and other multiple chronic diseases [10,11].

On the other hand, the influence of sarcopenia on quality of life (QoL) is a critical issue. For the elderly population, maintaining QoL is often regarded as just as crucial as longevity. Previous studies have explored the impact of sarcopenia on QoL using tools like the EuroQol-5 dimension (EQ-5D) [12], the Short-Form 36-Item (SF-36) Questionnaire [13,14] or the Sarcopenia Quality of Life (SarQoL) questionnaire [15] and showed significantly lower HRQoL in sarcopenic patients [16]. However, most studies have treated sarcopenia as a binary diagnosis (presence vs. absence) and there is only a few studies focusing on the impact of the severity of sarcopenia, which revealed heterogeneous results [13,17]. As sarcopenia is defined as a progressive disorder, it remains unclear whether the decline in QoL occurs immediately upon the onset of muscle mass loss, or if it is precipitated only when the condition progresses to a more severe stage involving functional impairment. Furthermore, the 2019 AWGS criteria explicitly categorize “severe sarcopenia” based on the presence of poor physical performance. Yet, there is a lack of in-depth discussion regarding how these different stages of severity specifically correlate with various domains of HRQoL. Understanding this relationship is vital for determining the optimal timing for clinical intervention. Therefore, the aim of this study is not only to evaluate the association between sarcopenia and HRQoL but specifically to investigate the impact of sarcopenia severity on QoL in the elderly population. We hypothesize that the severity of the condition plays a more pivotal role in QoL deterioration than the diagnosis alone.

## 2. Materials and Methods

### 2.1. Study Design and Participants

The Geriatric Medical Center of the Chang Gung Medical System, the largest medical system in Taiwan, has initiated a project to continuously enroll elderly patients from the outpatient department to establish a comprehensive cohort addressing several major geriatric issues, including sarcopenia, cognitive impairment and depression. Based on this project, this study enrolled elderly patients aged 65 and older from the geriatric outpatient department at Chang Gung Memorial Hospital, Linkou branch. Patients who were unable to follow instructions, had a serious condition requiring hospitalization within the past year, or had a terminal disease—including malignancies or severe dementia—were excluded. Clinical information, including gender, age, body mass index (BMI), body fat percentage, muscle mass, calf circumference, education level, religion, social activity and quality of life, was collected at the time of enrollment.

### 2.2. Sarcopenia and Quality of Life Assessment

**Figure 1:**
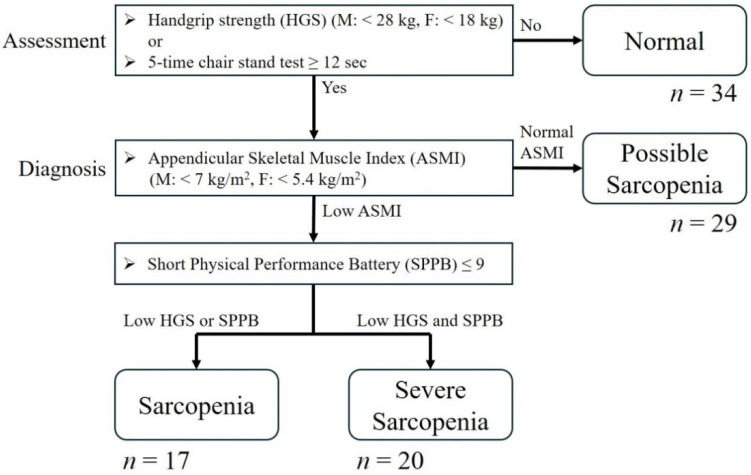
Algorithm of sarcopenia. The criteria were established by Asian Working Group for Sarcopenia (AWGS) in 2019. M: male. F: female.

As summarized in Figure 1, Sarcopenia was assessed in the enrolled participants according to the diagnostic criteria established by the AWGS in 2019 [5], which recommended diagnostic cutoffs of handgrip strength (HGS) < 28.0 kg for men and <18.0 kg for women as low muscle strength, while a 5-time chair stand time cutoff of ≥12 s as the cutoff for low physical performance to select possible sarcopenia candidate. To confirm diagnosis of sarcopenia, muscle mass is also required for evaluation. In this study, appendicular skeletal muscle index (ASMI) by dual-energy X-Ray absorptiometry (DXA) (Hologic Horizon W scanner) was used for muscle mass evaluation. The ASMI cutoffs for low muscle mass in sarcopenia diagnosis are as follows: <7.0 kg/m^2^ in men and <5.4 kg/m^2^ in women by DXA. A normal participant is defined as a participant without either low muscle strength or low physical performance by 5-time chair stand time. Possible sarcopenia is defined as normal muscle 5-time chair stand time. After diagnosed sarcopenia, severe sarcopenia is distinguished as combination of low muscle strength and low physical performance, which is defined as Short Physical Performance Battery (SPPB) score ≤ 9 by the AWGS 2019 recommendations [5]. Quality of life was assessed by The SF-36, rather than the SarQOL questionnaire, which is a generic health survey that uses 36 questions to measure functional health and wellbeing and was selected because the Chang Gung Geriatric Medical Center has established a comprehensive cohort addressing multiple major geriatric issues. Given its broader applicability, the SF-36 was adopted as a more general measure of quality of life. SF-36 measures eight domains: “Physical Functioning”, “Role limitation due to physical problems”, “Bodily Pain”, “General Health Perceptions”, “Vitality”, “Social Functioning”, “Role limitations due to emotional problems” and “Mental Health” [18].

### 2.3. Statistical Analysis

Quantitative variables were analyzed using the Kolmogorov–Smirnov test to assess their compatibility with a normal distribution. For normally distributed variables, one-way ANOVA was used to compare differences across sarcopenia status groups, whereas the Kruskal–Wallis test was applied to non-normally distributed variables. Categorical variables were compared using the chi-square test. Bonferroni corrections were applied for multiple comparisons in both quantitative and categorical variables. Pearson’s correlation coefficient was calculated to determine the relationship between these variables and aspects of quality of life. Significant variables were further examined using multivariable linear regression analysis. Data were analyzed using IBM SPSS Statistics version 26 for Windows (Armonk, NY, USA). A *p*-value of <0.05 was considered statistically significant.

## 3. Results

### 3.1. Baseline Characteristics

A total of 100 elderly participants were enrolled in this study and categorized into four groups based on the 2019 AWGS sarcopenia criteria: normal (n = 34), possible sarcopenia (n = 29), sarcopenia (n = 17) and severe sarcopenia (n = 20) (Figure 1).

The baseline characteristics according to sarcopenia status are presented in Table 1. Regarding physical characteristics, compared to the normal group, the severe sarcopenia group was significantly associated with older age (82.1 ± 7.1 vs. 71.7 ± 6.1, *p* < 0.05) and lower calf circumference (32.1 ± 2.7 vs. 35.8 ± 3.3, *p* < 0.05). Possible sarcopenia group showed a significantly increased BMI (29.5 ± 4.7 vs. 25.6 ± 3.6, *p* < 0.05) compared to the normal elderly group. Regarding body fat, education level, religion and social activity, no significant differences were observed between the two groups compared with the normal group.

### 3.2. Quality of Life for Different Severity of Sarcopenia

In terms of quality of life, the SF-36 questionnaire results for study participants across different severities of sarcopenia are presented in Table 2. We found that, compared to the normal group, the possible sarcopenia group had significantly lower scores in the domains of physical functioning and general health. However, no significant differences were found in any quality of life domains between the sarcopenia and normal groups.

Additionally, the severe sarcopenia group had significantly lower scores in all SF-36 quality of life domains compared to the normal group, except for social functioning and role-emotional. Furthermore, the severe sarcopenia group also showed significantly lower scores in physical functioning, general health and vitality compared to the sarcopenia group. The domain of social functioning and role-emotional showed no significance after Bonferroni correction.

### 3.3. The Correlation Between Potential Variables and Quality of Life

The correlation between potential baseline characteristics and the study participants’ quality of life is presented in Table 3. Most notably, the severity of sarcopenia showed a significant negative correlation with physical functioning, role-physical, bodily pain, general health, vitality and mental health. Additionally, participants’ age was significantly negatively correlated with physical functioning, role-physical and general health. In contrast, social activity showed a positive correlation with physical functioning, bodily pain, general health and vitality. Education was positively correlated only with physical functioning and general health, while being female was negatively correlated with vitality and mental health. Calf circumference showed a significant positive correlation with physical functioning, role-physical and vitality, whereas body fat was negatively correlated only with mental health. Height, BMI and religion showed no significant correlation with most quality of life domains.

A multivariate linear regression analysis was conducted to further evaluate the associations between potential variables and each domain of quality of life, as shown in Table 4. After adjustment, severe sarcopenia was significantly negatively associated with physical functioning, role-physical, bodily pain, general health, vitality and mental health. Possible sarcopenia was significantly associated with lower scores in physical functioning and general health. In addition, age was found to be significantly negatively correlated only with physical functioning, while body fat was negatively correlated only with mental health. Social activity showed a significant positive correlation with physical functioning and general health. Other variables did not show a significant correlation with any quality of life domains in the SF-36.

## 4. Discussion

In our study, the proportion of sarcopenia was 37% (35.5% in men and 38.1% in women), with 17% diagnosed as sarcopenia and 20% diagnosed as severe sarcopenia. In AWGS 2019 consensus, the review of epidemiology studies from Asian countries that used AWGS 2014 criteria, discovered that the prevalence of sarcopenia ranged from 5.5% to 25.7%, with male predominance (5.1–21.0% in men vs. 4.1–16.3% in women) [5]. There were also some studies of East Asia in recent years. The prevalence of sarcopenia in East Asia has mostly been reported in community-dwelling elderly populations; 41.0% in Korea (40.3% in men and 41.3% in women, respectively) [19] and 9.9% in Japan, (9.8% in men and 10.1% in women, respectively) [20]. In Taiwan, sarcopenia prevalence among community-dwelling elderly individuals has been reported to range from 3.9% to 16.6% (5.4–14.9% in men and 2.5–19% in women) [21,22,23,24] reaching approximately 50% in certain clinical settings such as daycare centers and hospitals [25,26]. Consistent with previous epidemiological studies in Asia, our findings fall within the expected range but lean towards a higher prevalence, likely due to the recruitment from a geriatric outpatient setting rather than the general community, where the enrolled participants were relatively healthy compared to those in daycare centers and hospitals, but have more comorbidities than community-dwelling elderly individuals.

Contrary to several prior studies in community or daycare centers that have proven that the confirmed sarcopenia is associated with poor quality of life [16,27,28,29], which simply compared sarcopenic and non-sarcopenic individuals, our results demonstrated that patients diagnosed with sarcopenia did not exhibit significantly poorer QoL compared to the normal group. A significant and comprehensive decline in HRQoL was observed only when the condition progressed to severe sarcopenia, which demonstrates poor scores across multiple domains, including physical functioning, role-physical, bodily pain, general health, vitality and mental health. This finding suggests that severe sarcopenia leads to a broader and more profound decline across nearly all aspects of daily life and may be considered a “threshold effect,” where the reduction in muscle mass and strength alone may be compensated for in daily living until physical performance is critically compromised. This aligns with the revised EWGSOP2 and AWSG 2019 consensus [3,5], which emphasizes that physical performance is the primary indicator of severity and the strongest predictor of adverse outcomes. Our data supports the notion that maintaining physical performance above a critical threshold may be a key to preserving HRQoL, even in the presence of muscle loss.

Interestingly, our study found that the possible sarcopenia group, characterized by lower muscle function but normal muscle mass, reported significantly lower scores in Physical Functioning and General Health compared to the normal group, performing even worse than the confirmed Sarcopenia group in some domain of HRQoL. The baseline characteristics reveal that this group had the highest BMI (29.5 ± 4.7 kg/m^2^) and body fat percentage (40.4 ± 6.7%). Previous studies have found that a higher BMI can be a protective factor against sarcopenia [30,31,32] but BMI exceeding the cutoff for obesity is still one of the important factors that lead to further sarcopenia by increasing the level of pro-inflammatory factors, promoting insulin resistance [33]. Fat mass increases with aging and can gradually infiltrate into skeletal muscle, resulting in changes in muscle fiber structure and leading to loss of skeletal muscle mass, strength and function [34,35]. One recent study which enrolled Chinese community-dwelling older adults has demonstrated that increased body fat mass is an associated risk factor of sarcopenia [36]. In addition, the phenotype of our possible sarcopenia group strongly resembles “Dynapenic Obesity”, a condition combining low muscle strength (dynapenia) with obesity [37]. Previous research indicated that dynapenic obesity leads to greater functional impairment and disability than either sarcopenia or obesity alone, likely due to the “double burden” of carrying excess weight with insufficient muscle force [38,39]. Our findings suggest that patients with normal muscle mass but low strength and high body fat, whose functional QoL may be already significantly compromised, should not be overlooked in clinical practice.

Beyond sarcopenia status, there are several interesting covariates that demonstrated significant associations with HRQoL which merit further discussion. First, our multivariate analysis revealed that body fat percentage is not only associated with sarcopenia but is also an independent correlate of mental health after adjusting for potential confounders. This finding aligns with previous studies indicating that elevated body fat percentage is strongly associated with higher prevalence [40] and also increased risk of depression [41,42]. However, the complex interplay between adiposity and mental health in older adults warrants further validation through large-scale longitudinal studies. Second, older age is generally regarded as the most important factor for worse quality of life and several prior studies also indicated this point [13,35]. However, in this study, after adjusting for other crucial variables in the regression analysis, age remained significantly associated with the physical functioning domain. These findings suggest that, in this study population, the level of physical performance exhibits a stronger statistical association with overall HRQoL than chronological age alone.

Finally, our study reaffirmed the protective role of social activity, which was positively associated with Physical Functioning and General Health. As for social activity, it is seen as a resource for older people to fulfil their needs, in turn influencing their quality of life. Increased number of social activities have also found to have a positive association with an increase in health-related quality of life [43]. Social participation is an important health behavior for the health and quality of life outcomes among older adults [44], which suggested that social participation buffers against functional decline and enhances perceived health status.

### Limitation

Our study has some limitations that should be acknowledged. First, the cross-sectional design precludes causal inferences between sarcopenia severity and HRQoL. Second, the relatively small number of participants limited statistical power and subgroup analyses. Although primary findings remained significant after Bonferroni correction, the absence of a pre hoc power calculation suggests that the results should be interpreted with caution. Third, recruitment from a tertiary geriatric outpatient clinic introduces selection bias. Our cohort likely possesses a higher comorbidity burden than community-dwelling older adults, limiting generalizability to the broader healthy elderly population. Fourth, we acknowledge potential collinearity among age, sarcopenia severity and adiposity. Significant differences in baseline characteristics across study groups was also noted, and while multivariate models were adjusted for these factors, their biological interrelation suggests residual confounding may persist. Fifth, the use of the generic SF-36, rather than the sarcopenia-specific SarQoL, may have diluted the specific impact of muscle mass loss on quality of life. Finally, the absence of objective functional measures, such as Activities of Daily Living (ADL) or Instrumental Activities of Daily Living (IADL) is a limitation, although the SF-36 Physical Functioning domain served as a subjective proxy to assess physical limitations. The inclusion of objective functional disability scales would have provided additional context regarding the participants’ independence. We plan to design a longitudinal study in the future as part of our ongoing project with larger sample sizes and comprehensive functional assessments to validate our findings and clarify the causal pathways.

## 5. Conclusions

In summary, our results found that the relationship between sarcopenia and QoL is not linear. Severe sarcopenia, rather than sarcopenia, is associated with widespread HRQoL deterioration, while possible sarcopenia with obesity represents a unique phenotype with specific functional deficits. These distinctions may suggest the necessity of a stage-specific management strategy to restore not only physical performance but also quality of life. We look forward to further studies focusing on the risk factors, prevention and interventions for patients with varying degrees of sarcopenia.

## Figures and Tables

**Table 1:** Characteristics of study participants.

Characteristics	Total (n = 100)	Normal (n = 34)	Possible Sarcopenia (n = 29)	Sarcopenia (n = 17)	Severe Sarcopenia (n = 20)	Significance ^a^
Gender, n (%)						N
Male	45 (45.00)	18 (52.94)	11 (37.93)	8 (47.06)	8 (40.00)	
Female	55 (55.00)	16 (47.06)	18 (62.07)	9 (52.94)	12 (60.00)	
Physical Characteristics, mean ± S.D.						
Age, y/o	75.2 ± 7.2	71.7 ± 6.1	74.3 ± 5.9	75.9 ± 6.2	82.1 ± 7.1 *	
Height, cm	157.7 ± 8.4	159.7 ± 7.6	157.5 ± 8.3	156.3 ± 9.7	155.7 ± 8.2	
BMI, kg/m^2^	26.0 ± 4.5	25.6 ± 3.6	29.5 ± 4.7 *	24.6 ± 2.4	22.8 ± 3.6	
Body Fat, %	37.8 ± 6.5	35.8 ± 6.4	40.4 ± 6.7	38.3 ± 6.2	36.8 ± 5.9	
Calf Circumference, cm	34.9 ± 3.5	35.8 ± 3.3	36.7 ± 3.3	33.4 ± 2.2	32.1 ± 2.7 *	
Education, n (%)						N
≥High School	39 (39.0)	19 (55.9)	9 (31.0)	8 (47.1)	3 (15.0)	
<High School	61 (61.0)	15 (44.1)	20 (69.0)	9 (52.9)	17 (85.0)	
Religion, n (%)						N
Yes	77 (77.0)	27 (79.4)	22 (75.9)	13 (76.5)	15 (75.0)	
No	23 (23.0)	7 (20.6)	7 (24.1)	4 (23.5)	5 (25.0)	
Social Activity, n (%)						N
Yes	77 (77.00)	31 (91.18)	21 (72.41)	13 (76.47)	12 (60.00)	
No	23 (23.00)	3 (8.82)	8 (27.59)	4 (23.53)	8 (40.00)	

^a^ Significant difference was positive (P) or negative (N) in chi-square test with Bonferroni correction among normal, possible sarcopenia, sarcopenia and severe sarcopenia. * Significant difference was observed after Bonferroni correction when compared with normal.

**Table 2:** Quality of life of study participants.

SF-36	Normal (n = 34)	Possible Sarcopenia (n = 29)	Sarcopenia (n = 17)	Severe Sarcopenia (n = 20)
Physical Functioning	92.7 ± 10.6	72.1 ± 25.4 *	83.2 ± 18.6	43.4 ± 31.8 *^, +^
Role-Physical	96.3 ± 17.6	87.9 ± 24.7	80.9 ± 34.8	54.0 ± 50.2 *
Bodily Pain	88.8 ± 17.3	81.1 ± 26.2	85.7 ± 25.5	63.6 ± 31.1 *
General Health	82.4 ± 16.0	67.1 ± 22.1 *	79.5 ± 20.8	49.1 ± 28.1 *^, +^
Vitality	77.7 ± 14.7	73.1 ± 16.3	74.1 ± 18.1	56.5 ± 18.9 *^, +^
Social Functioning	96.3 ± 12.9	97.0 ± 9.8	97.8 ± 9.1	83.6 ± 29.2
Role-Emotional	93.1 ± 21.4	96.6 ± 13.6	96.1 ± 16.2	77.2 ± 36.9
Mental Health	87.3 ± 12.2	80.6 ± 16.7	86.1 ± 14.6	74.2 ± 18.5 *

All items were displayed as mean ± S.D. Role-physical: role limitation due to physical problems. Role-emotional: role limitation due to emotional problems. Significant difference was observed after Bonferroni correction when compared with normal (*) or sarcopenia (^+^).

**Table 3:** Potential variables associated with study participants’ quality of life.

SF-36	Pearson’s Correlation Coefficient (r)									
	Sarcopenia a	Gender b	Age	Height	BMI	Body Fat	Calf Circumference	Education c	Religion d	Social Activity e
Physical Functioning	−0.546 *	−0.142	−0.477 *	0.196	0.003	−0.158	0.293 *	0.237 *	0.149	0.334 *
Role-Physical	−0.425 *	−0.156	−0.240 *	0.183	0.048	−0.200	0.214 *	0.052	−0.080	0.166
Bodily Pain	−0.301 *	−0.073	−0.034	0.095	0.114	0.029	0.103	0.133	−0.075	0.272 *
General Health	−0.406 *	−0.093	−0.336 *	0.070	0.000	−0.099	0.176	0.225 *	0.092	0.346 *
Vitality	−0.378 *	−0.214 *	−0.166	0.120	0.116	−0.139	0.255 *	0.127	−0.060	0.243 *
Mental Health	−0.244 *	−0.257 *	−0.106	0.191	−0.059	−0.354 *	0.195	0.007	−0.117	0.075

a normal, possible sarcopenia, sarcopenia and severe sarcopenia were represented as 0, 1, 2 and 3; b male and female were represented as 0 and 1; c education level < high school and ≥high school were represented as 0 and 1; d with and without religious faith were represented as 1 and 0; e with and without social activity were represented as 1 and 0 in analyses. Role-physical: role limitation due to physical problems. Role-emotional: role limitation due to emotional problems. * Statistical significance was observed.

**Table 4:** Relationships between variables and study participants’ quality of life.

SF-36	Variables	Multivariable Linear Regression			
		B	95% CI		*p*-Value
Physical Functioning	Possible Sarcopenia	−16.7	−28.1	−5.4	0.004
	Sarcopenia	−1.7	−15.1	11.8	0.807
	Severe Sarcopenia	−33.0	−48.7	−17.2	0.000
	Age	−0.8	−1.6	−0.1	0.022
	Calf Circumference	0.9	−0.5	2.4	0.206
	Education ^a^	−0.3	−9.6	9.0	0.949
	Social Activity ^b^	11.2	0.4	22.0	0.043
Role-Physical	Possible Sarcopenia	−9.2	−25.6	7.2	0.269
	Sarcopenia	−15.8	−35.7	4.1	0.118
	Severe Sarcopenia	−42.6	−65.2	−20.1	0.000
	Age	0.0	−1.1	1.1	0.983
	Calf Circumference	0.1	−2.0	2.3	0.906
Bodily Pain	Possible Sarcopenia	−5.5	−17.8	6.8	0.374
	Sarcopenia	−1.4	−15.7	12.9	0.851
	Severe Sarcopenia	−21.3	−35.6	−7.1	0.004
	Social Activity ^b^	11.7	−0.1	23.6	0.053
General Health	Possible Sarcopenia	−11.6	−22.5	−0.6	0.038
	Sarcopenia	0.6	−12.1	13.3	0.922
	Severe Sarcopenia	−25.1	−39.1	−11.1	0.001
	Age	−0.3	−1.0	0.3	0.318
	Education ^a^	1.5	−7.7	10.6	0.751
	Social Activity ^b^	12.7	2.4	23.1	0.017
Vitality	Possible Sarcopenia	−2.4	−10.9	6.2	0.582
	Sarcopenia	−0.5	−10.6	9.7	0.924
	Severe Sarcopenia	−15.6	−26.2	−5.1	0.004
	Gender ^c^	−6.5	−13.2	0.1	0.055
	Calf Circumference	0.6	−0.6	1.7	0.323
	Social Activity ^b^	7.7	−0.5	16.0	0.067
Mental Health	Possible Sarcopenia	−3.3	−11.0	4.4	0.394
	Sarcopenia	0.9	−7.7	9.6	0.829
	Severe Sarcopenia	−12.3	−20.6	−4.0	0.004
	Gender ^c^	−0.6	−8.1	6.8	0.867
	Body Fat	−0.8	−1.4	−0.2	0.006

^a^ Education level < high school and ≥high school were represented as 0 and 1; ^b^ with and without social activity were represented as 1 and 0; ^c^ male and female were represented as 0 and 1 in analyses. Role-physical: role limitation due to physical problems.

## Data Availability

The datasets used and analyzed during the current study available from the corresponding author on reasonable request.

## Abbreviations

The following abbreviations are used in this manuscript:

QoL	Quality of life
HRQoL	Health-related quality of life
EWGSOP	European Work Group on Sarcopenia in Older people
AWGS	Asian Working Group for Sarcopenia
SF-36	36-Item Short Form Survey
BMI	Body mass index
HGS	Handgrip strength
ASMI	Appendicular skeletal muscle index
DXA	dual-energy X-Ray absorptiometry
